# The global profile of individuals undergoing total knee replacement surgery through a PROGRESS-PLUS equity lens: A systematic review

**DOI:** 10.4102/sajp.v82i1.2303

**Published:** 2026-02-27

**Authors:** Marisa Coetzee, Amanda Clifford, Dominique C. Leibbrandt, Jacobus Jordaan, Quinette Louw

**Affiliations:** 1 Department of Physiotherapy, Faculty of Health and Rehabilitation Sciences, Stellenbosch University, Cape Town, South Africa; 2 School of Allied Health, Health Research Institute, Ageing Research Centre, University of Limerick, Limerick, Ireland; 3 Department of Orthopaedic Surgery, Faculty of Medicine and Health Sciences, Stellenbosch University, Cape Town, South Africa

**Keywords:** osteoarthritis, total knee replacement, PROGESS-Plus, health equity, patient profile, rehabilitation, physiotherapy

## Abstract

**Background:**

Osteoarthritis (OA) of the knee is a common, disabling condition influenced by multiple biopsychosocial factors and often requiring a total knee replacement (TKR). However, most rehabilitation programmes are developed in high-income countries, potentially limiting transferability to lower-income settings with distinct health equity challenges.

**Objectives:**

This study aimed to describe the demographic and health equity profiles of adults undergoing TKR for primary OA across low-, middle- and high-income countries.

**Method:**

A systematic literature search was conducted in PubMed, Scopus, EBSCOhost, Web of Science and ProQuest for peer-reviewed primary research, including observational studies, randomised controlled trials and grey literature published between 2020 and 2024. Studies reporting on individuals undergoing TKR were selected. Data extraction followed the PROGRESS-Plus framework, and a descriptive synthesis of demographic and health equity information was performed.

**Results:**

The review included 101 studies with over 3.2 million participants, predominantly from high- and upper-middle-income countries, with no representation from Africa. Although females consistently represented the majority (54% – 86%), key health equity indicators such as socioeconomic status and education were inadequately reported. Clinical characteristics also varied, with a higher mean BMI observed in high-income country studies. Substantial methodological heterogeneity precluded meta-analysis.

**Conclusion:**

There is extensive global research on TKR; however, data from lower-income countries is scarce, and health equity factors are poorly reported.

**Clinical implications:**

Inconsistent reporting of outcome measures and limited reporting of health equity in global studies limit the implementation of rehabilitation programmes in low-resource settings. These settings would benefit from detailed equity data to adapt interventions to local patient needs. In addition, better integration of social determinants of health into physiotherapy practice can enhance personalised care and fair resource distribution.

## Introduction

Osteoarthritis (OA) most frequently affects the knee joint. While its estimated global prevalence is 22% in individuals over 40 years of age (Cui et al. 2020), the condition predominantly impacts older adults, and its global, regional and national burden continues to rise significantly, with analyses projecting this burden to 2050 (Steinmetz et al. 2023).

Furthermore, the burden of OA is escalating, particularly among postmenopausal women globally. The classification of knee OA is typically demarcated into two main categories: primary OA, which lacks a clear causal factor for articular degeneration, and secondary OA, which is associated with specific factors such as joint trauma (e.g. previous fractures, ligament and meniscus injuries) or inflammatory conditions such as rheumatoid arthritis (Hsu & Silwec 2023).

The risk factors for the development of primary knee OA include age, gender, genetics, increased body mass index (BMI), physical activity levels and occupational demands (Cui et al. 2020). Additionally, factors that affect both the clinical progression (level of pain, functional ability) and the structural progression of knee OA also include socioeconomic indicators (e.g. level of education and social class), psychological factors (coping, mechanisms, anxiety, depressive states) and the presence of comorbidities (Bastick et al. 2016; Chen et al. 2025; Deveza, Loeser & Katz 2017; Li et al. 2024; Silverwood et al. 2015; Swinnen et al. 2021). Many of these risk factors are interconnected within the social context of the individual, and in conjunction with personal factors, community perceptions and psychological influences, they exert a considerable influence on the health-related outcomes for individuals presenting with knee OA (Luong, Lohmander & Sowers 2012). An important aspect of managing primary knee OA is the identification of modifiable risk factors, as these can be more effectively addressed through targeted interventions (Georgiev & Angelo 2019).

Given the complex and diverse presentation of knee OA, management strategies should consider the individual within their biopsychosocial context. However, a significant limitation is that data exploring risk factors, social determinants and progression trajectories primarily come from high-income countries (HICs). These data may not accurately reflect the profiles of individuals experiencing knee OA in low-middle-income countries (LMICs), where a significant proportion of the population faces lower socioeconomic circumstances (Dell’ Isola et al. 2016, 2018; Deveza et al. 2017; Li et al. 2024; Luong et al. 2012; Steinmetz et al. 2023). Existing international OA management interventions are typically designed for HIC contexts and may therefore not be generalisable or transferable to local contexts in LMIC because of health equity differences. Health equity differences may introduce barriers to the implementation of best-evidence OA care such as fragmented healthcare systems, inadequate human resources and inexperience among health professionals in managing OA post TKR (Castro et al. 2021; Keller & Sankah 2025).

Evidence suggests that physiotherapy plays a vital role in addressing the complex biopsychosocial factors following a TKR, as physiotherapists possess specialised expertise in understanding the multifaceted contributors to chronic pain and functional limitations (Kohia et al. 2015; Nishimoto et al. 2025). However, the availability and scope of physiotherapy services may vary considerably between high-income and LMICs, potentially creating additional barriers to comprehensive post-TKR rehabilitation.

To develop effective, context-specific programmes, the global demographic profile of individuals with OA should be designed using an equity lens that considers key factors influencing health outcomes. The PROGRESS-Plus equity lens framework is an acronym representing the following key factors: place of residence, race or ethnicity or culture or language, occupation, gender or sex, religion, education, socioeconomic status (SES), social capital, age, disability, sexual orientation and other vulnerable groups (Kavanagh, Oliver & Lorenc 2008). This framework was developed for description and assessment of social determinants related to health equity across populations, offering insights for translating interventions into different contexts (Kavanagh et al. 2008), by including descriptors linked to the variability in health outcomes (O’ Neill et al. 2014).

To the authors’ knowledge, no studies have comprehensively described the global demographic and social profiles of individuals undergoing TKR. An overview of these profiles through an equity-focused lens, such as the PROGRESS-Plus framework, can facilitate the adaptation of existing rehabilitation interventions to diverse local contexts. Therefore, this systematic review aims to describe the demographic characteristics and PROGRESS-Plus profiles of adults undergoing TKR for primary knee OA across low-, middle- and high-income countries. The findings are intended to identify evidence-based interventions and highlight critical gaps in research from lower-income contexts, particularly African settings. This will inform future research priorities and adapted implementation strategies for underrepresented contexts.

## Research methods and design

### Eligibility criteria

The systematic review used specific criteria for the inclusion of studies, as detailed in Table 1. The eligibility criteria for this systematic review included studies involving adults (≥ 18 years) awaiting or undergoing TKR surgery for primary knee OA. Eligible study designs comprised observational studies (cross-sectional, cohort, case-control) and randomised controlled trials. Studies were required to recruit participants sequentially or consecutively, with minimal age restrictions permissible. Only studies reporting baseline pre-surgical information specific to knee OA were included. To maintain feasibility and relevance, the review was limited to studies published between 2020 and 2024 because of the vast number of publications on the topic. Studies focusing on patellofemoral joint OA, secondary OA because of trauma or primary OA diagnoses not related to TKR individuals were excluded. Case studies and systematic reviews were excluded. Studies with restricted age ranges or those that selectively excluded participants based on factors such as BMI or comorbidities were not considered. Additionally, studies presenting combined hip and knee data, duplicate cohorts or those superseded by a more comprehensive included study were excluded. Studies published outside the specified 5-year period were also excluded to ensure manageability, given the volume of literature.

**TABLE 1:** Eligibility criteria for the inclusion of studies in the systematic review.

Category	Inclusion	Exclusion
Population	Adults Awaiting and/or undergoing primary TKR Tibiofemoral joint OA Primary OA	Patellofemoral joint OA Secondary OA (trauma-related)
Study design	Observational studies (cross-sectional, cohort, case-control designs) Randomised controlled trials (RCT’s).	Case studies Reviews
Recruitment	All participants were included sequentially and/or consecutively Minimal restrictions on the age limit	Restricted age range (example, between 50 years and 70 years) Recruitment did not exclude any individuals based on strict criteria such as BMI, comorbidities, etc.
Type of information	Studies reporting demographic and social profile information Had to report on the baseline pre-surgical information of knee OA Grey literature (theses and published conference presentations)	Data presented as combined for hip and knee Studies reporting on the exact same cohort of individuals (the publication/study with the most comprehensive reporting of the PROGRESS-Plus factors was included)

RCT, Randomised controlled trials; OA, osteoarthritis; BMI, body mass index; TKR, total knee replacement; PROGRESS, place of residence, race or ethnicity or culture or language, occupation, gender or sex, religion, education, socioeconomic status, social capital.

For the purpose of our review, ‘undergoing Total Knee Replacement (TKR)’ refers to individuals who are either awaiting TKR surgery or have already received the procedure, provided that baseline pre-surgical information is reported. Classification of countries by income group was based on the World Bank’s List of Income Classification of Economies (available at www.worldbank.org/en/home), which categorises economies according to the Atlas Gross National Income (GNI) per capita into four groups: low-income, lower-middle-income, upper-middle-income and high-income countries. Primary OA was defined as articular degeneration occurring without any identifiable underlying cause (Hsu & Siwec 2023). In contrast, secondary OA was considered to result from either abnormal mechanical loading of the joint, such as post-traumatic events, or conditions affecting normal articular cartilage (Hsu & Siwec 2023).

### Search strategy

In collaboration with a faculty librarian who was experienced in conducting systematic searches, an initial exploratory search was conducted using the Stellenbosch University online library to identify databases containing relevant peer-reviewed literature, as well as grey literature, including academic theses. The following databases, PubMed (Medline), Scopus (abstracts from Elsevier or other sources), EBSCOhost (Africa Wide, CINAHL, Academic Premier, Health Source Nursing), Web of Science and ProQuest, were accessed for our review. Key search terms were identified by reviewing author keywords and index terms in relevant articles retrieved from PubMed and the Cochrane Library. A full list of these terms is provided in Online Appendix 1 – Table 1-A1, and the specific search strings used for each database are detailed in Online Appendix 1 – Table 2-A1.

### Study selection and procedure

Following the database searches outlined in Online Appendix 1 – Table 2-A1, the identified studies were exported as comma-separated values (CSV) files containing the titles and abstracts. These CSV files were imported into the Rayyan Intelligent Systematic Review web-based software (https://www.rayyan.ai/) where automated duplicate detection was performed by the software. After duplicates were removed, the remaining titles were screened using the eligibility criteria. Articles not meeting the criteria based on their titles were excluded, after which the abstracts of the remaining studies were assessed for relevance and potential inclusion. Full-text versions of eligible articles were retrieved for further review by the main author and research assistant, and where conflicts arose, the supervisory team were consulted.

**FIGURE 1:**
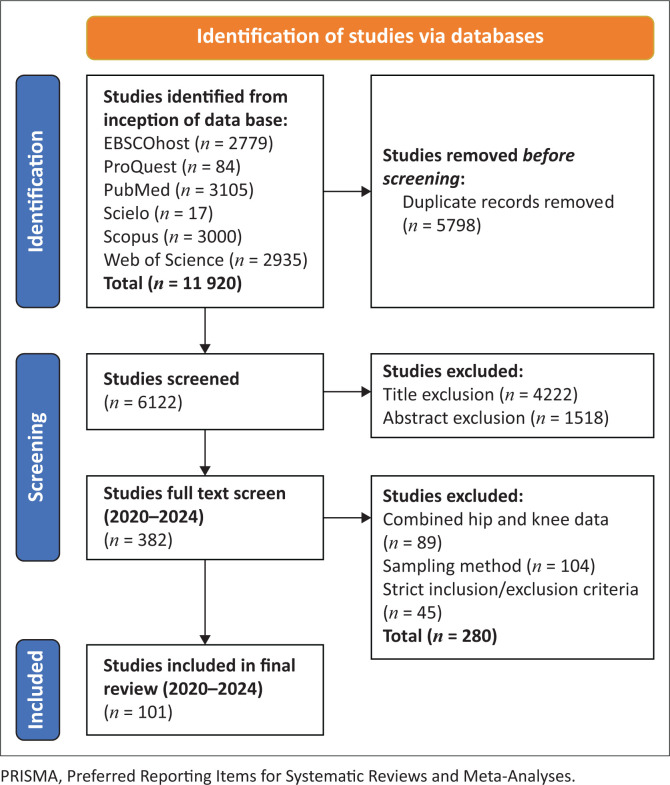
Identification of included studies according to the PRISMA 2020 flow diagram for new systematic reviews.

Both the primary researcher and a research assistant independently reviewed the titles, abstracts and full texts according to the eligibility criteria. Upon selection of the final studies, the supervisory team reviewed the articles and the extracted data. Any conflicts were resolved by the supervisory team. Reporting of the review process was done according to the Preferred Reporting Items for Systematic Reviews and Meta-Analyses (PRISMA) 2020 flow diagram for new systematic reviews, which included database and register search results (Page et al. 2021). This flowchart can be seen in Figure 1 in the results section.

### Assessment of reporting quality

Reporting quality of the final list of included studies was assessed using the appropriate study design appraisal tool from the Strengthening the Reporting of Observational Studies in Epidemiology (STROBE) checklist (Von Elm et al. 2007) or the JBI Critical Appraisal Checklist for Randomised Controlled Trials (Tufanaru et al. 2020). The focus on assessing the reporting quality of the included studies was prioritised because of the descriptive nature of the data sought and to understand the profile of participants, especially in observational studies, where quality reporting is essential. Because of time constraints, two research assistants were educated on using the STROBE checklist by the primary researcher and conducted the quality of reporting assessment, each taking half of the studies. All uncertainties and queries were resolved by the primary researcher. The STROBE checklist is presented in Online Appendix 1 – Table 3-A1.

### Data extraction and management

In addition to the study characteristics (which included title, authors, date of publication, aims and objectives, study design, study setting, sample size and results), the PROGRESS Plus framework was used as a guide to extract the relevant information required for applying an equity lens. This included place of residence, race or ethnicity or culture or language, occupation, gender or sex, religion, education, SES, social capital, age, disability and other vulnerable groups (Dell’Isola & Steultjens 2018). Furthermore, smoking, BMI and severity of OA were also extracted for descriptive purposes. Data were extracted by the main reviewer into a Microsoft Excel spreadsheet for recordkeeping and analysis, and a description of the factors can be seen in Table 2.

**TABLE 2:** The PROGRESS-Plus factors assessed and the description of the extracted data.

Factor	Title in table	Description of data extracted
Place of residence	Country	Country and income level according to worldbank.org
Race or ethnicity	Race	Number of people listed as white
Occupation	Occupation	Number of people showed as currently employed or on pension
Gender	Gender	Number of female participants
Religion	n/a	None of the studies reported in this
Education	Education	Number of people having finished secondary education or higher
Socioeconomic	SES	Number of people listed as lower on the socioeconomic scale
Social capital	Social capital	Number of people listed as married, with a partner or having support
Age	Age	Mean age and range
Lifestyle	BMI Smoking	Body mass index, mean and s.d. Number of people who are smokers
Disability	OA severity	Number of people scoring Level four on the Kellgren–Lawrence scale for radiographic severity of OA

OA, osteoarthritis; BMI, body mass index; SES, socioeconomic status; s.d., standard deviation; n/a, not applicable.

### Data synthesis and reporting

Basic synthesis of the data was done using the descriptive quantitative information available from the studies. Most of the studies reported the demographic and social data using percentages, averages and ranges. Data are reported in the ranges provided by the studies, and counts were combined as a total and percentages calculated according to the total counts of the combined studies.

### Ethical considerations

This systematic review was registered with the International Prospective Register of Systematic Reviews (PROSPERO) (review number 284634 on https://www.crd.york.ac.uk/prospero/) and adhered to the Preferred Reporting Items for Systematic Reviews and Meta-Analysis (PRISMA) Statement (Page et al. 2021). Our study was approved by the Stellenbosch University Health and Research Ethics Committee under Ethics number S20/11/315. The protocol was published and can be accessed online on https://hdl.handle.net/10520/ejc-sajp_v78_i1_a1649 (Coetzee et al. 2022).

## Results

The systematic review identified a total of 101 studies (Figure 1) from diverse global regions, encompassing East Asia and the Pacific (*n* = 23), Europe and Central Asia (*n* = 38), the Middle East (*n* = 3), North America (*n* = 33) and South Asia (*n* = 4). Of these, only five were conducted in lower middle-income countries, specifically the Philippines and India (Bakshi et al. 2021; Chaudhary et al. 2024; Chhaya, Padmashri & Krishnan 2020; Devasenpathy et al. 2020; Dumlao, Delgado & Azores 2020), and nine in upper middle-income countries (Bian et al. 2021; Bradbury et al. 2024; Chan et al. 2020; Kaya, Seyman & Kaya 2024; Kocic et al. 2024; Liu et al. 2020, 2021; Moghtadaei et al. 2020; Wang et al. 2022). The rest were conducted in HICs. No African studies were included. Detailed information regarding the selected articles is presented in Table 3, segmented by geographic region.

**TABLE 3:** Study characteristics and participant information from the included studies according to region.

Article identifier	Aim	Data source	Study design	Instruments	Country	Income	Sample size
**Articles from the East Asia and Pacific region (*n* = 23)**							
Heath et al. (2021)	Describe the pre- and postoperative self-reported health and quality of life status.	National registry	Retrospective cohort	EQ-5D-5L; OKS; KOOS-12	Australia	High income	8299
Genel et al. (2024)	Determine the proportion of TKA and THA patients who use prescribed opioids regularly (daily) before surgery (i.e. opioid use reported between the time of waitlisting and any time up to 3 months before surgery).	National and regional registries	Retrospective cohort	OKS; EQ-VAS	Australia	High income	1187
Johns et al. (2024)	Determine the relative risk (RR) of having high pain post-op and evaluate the association of high pain on function.	Public and private hospital databases	Retrospective cohort	OKS; EQ-VAS	Australia	High income	718
Liu et al. (2020)	Determine mental health before TKA, preoperative psychological intervention would be necessary.	Hospital database	Prospective observational	WOMAC; SF-36	China	Upper middle income	532
Wang et al. (2022)	Assess if the educational level of patients will affect the functional recovery after total knee arthroplasty (TKA).	Hospital database	Retrospective cohort	HSS; WOMAC; SF-12	China	Upper middle income	334
Bian et al. (2021)	Examine the prevalence of preoperative psychological distress and its influences on patient satisfaction and function after TKA.	Hospital database	Prospective cohort	KSS	China	Upper middle income	210
Liu et al. (2021)	Assess the prevalence of patient satisfaction after TKA and the roles of different scales in measuring overall patient satisfaction.	Hospital database	Retrospective cohort	WOMAC; KSS; (SF)-12	China	Upper middle income	545
Chan et al. (2020)	Determine the prevalence of prediabetes and diabetes in patients who underwent TKA and whether universal HbA1c screening and glycaemic control affected the rate of PJI after TKA.	Hospital database	Cross-sectional	-	Hong Kong SAR, China	High income/ upper middle income (China)	1566
Tanaka et al. (2022)	To determine if self-reported physical activity was related to muscle strength and walking ability in patients with knee osteoarthritis awaiting total knee arthroplasty.	Hospital databases	Cross-sectional	-	Japan	High income	767
Hasegawa et al. (2021)	To evaluate the presence of neuropathic pain in the knees of OA patients using the painDETECT questionnaire (PDQ).	Hospital database	Prospective cohort	-	Japan	High income	158
Tobinaga et al. (2021)	Examined the impact of physical activity SE on health-related quality of life (HRQOL) alongside other factors.	Hospital database	Cross-sectional	WOMAC; SF-36v2	Japan	High income	106
Nagira et al. (2022)	Explore the trends in patient characteristics and implant survivorship (IS) for primary total knee arthroplasty (TKA) over the past three decades.	Hospital database	Retrospective cohort	-	Japan	High income	307
Choi et al. (2020)	Investigate the association of factors for OA-induced knee pain in Taiwanese patients who received total knee replacements (TKRs).	Hospital database	Retrospective cross-sectional	-	Korea, Rep.	High income	158
Sook Joung et al. (2020)	Identify preoperative physical performance factors that predict stair-climbing ability at 1 month after total knee arthroplasty.	Hospital database	Prospective cohort	WOMAC; EQ-5D	Korea, Rep.	High income	213
Sauder et al. (2020)	Investigate differences in patient demographics between centres in the United States, Scandinavia and South Korea for patients undergoing primary TKA.	Hospital databases	Cross-sectional	KOOS	Korea, Rep.	High income	100
Kim et al. (2021)	To determine the effect of comorbidities on the physical function and quality of life of patients at 3 months after total knee arthroplasty.	Hospital database	Retrospective cohort	WOMAC; EQ-5D	Korea, Rep.	High income	140
Baek et al. (2024)	Investigated the prevalence of adrenal insufficiency among patients in Korea admitted for TKA.	Hospital database	Prospective cross-sectional	-	Korea, Rep.	High income	200
Shon, Kim and Jo (2024)	Compare sex differences in the incidence of sarcopenia in patients undergoing TKA for advanced knee osteoarthritis (OA).	Hospital database	Retrospective comparative	KOOS; WOMAC; (SF)-12	Korea, Rep.	High income	892
Snell et al. (2021)	Examined associations between self-reported and clinician-assessed comorbidity and quality of life (QOL) outcomes after hip and knee replacement.	National registry	Cross-sectional	EUROHIS-QOL; OKS	New Zealand	High income	214
Dumlao et al. (2021)	Describe the demographics, clinical profiles and outcomes of arthroplasty patients.	Hospital database	Descriptive and retrospective review	-	Philippines	Lower middle income	111
Zhang et al. (2021)	Compare the predictive performance of machine learning (ML) algorithms and preoperative PROM thresholds in predicting minimal clinically important difference (MCID) attainment at 2 years after TKA.	National registry	Prospective cohort	WOMAC; SF-36	Singapore	High income	2840
Sim et al. (2024)	Evaluate the impact on knee function and quality of life of patients who had their planned TKA postponed because of the pandemic.	Hospital database	Retrospective review	KSFS; KSS; OKS; SF-36	Singapore	High income	160
Chio et al. (2020)	Investigate the association factors for OA-induced knee pain in Taiwanese patients who received total knee replacements (TKRs).	Hospital database	Retrospective cohort	-	Taiwan, China	High income	357
**Articles from the Europe and Central Asia region (*n* = 38)**							
Neuprez et al. (2020)	Determine if benefits observed during the first year were maintained or improved over time and to clarify their determinants.	Hospital database	Prospective cohort	SF-36; WOMAC; EQ-VAS; EQ-5D	Belgium	High income	280
El-Galaly et al. (2020)	Can a clinically meaningful model be built on the preoperative factors included in the Danish Knee Arthroplasty Registry.	National registry	Retrospective review	KSS;	Denmark	High income	25 104
Justesen et al. (2020)	Investigate whether intraoperative contamination results in lower patient-reported outcomes (PRO) for patients.	Hospital databases	Prospective cohort	OKS; FJS; EQ-5D-5L; EQ-VAS	Denmark	High income	630
Larsen et al. (2020)	Assess the changes in pain intensity and functional capacity in patients with post-surgical complications after 3 weeks of rehabilitation.	Hospital database	Retrospective cohort	KOOS;	Denmark	High income	166
Sauder et al. (2020)	Investigate differences in patient demographics between centres in the United States, Scandinavia and South Korea for patients undergoing primary TKA.	Hospital databases	Cross-sectional	KOOS	Denmark	High income	169
Bradbury et al. (2024)	Compare outcomes between RPT and OPT in patients undergoing SDD 36 TKA.	Hospital database	RCT	VR-12; KOOS, Jr;	Georgia	Upper middle income	197
Green, Walsh and Al-Dadah (2023_)_	Compare the clinic outcomes of THR and TKR using a comprehensive range of patient-reported outcome measures (PROMs).	Hospital database	Prospective longitudinal observational	EQ-5D; SF-12; WOMAC; KOOS; OKS	Germany	High income	63
Siviero et al. (2020)	Investigate patient QOL at the time of knee replacement surgery and 3 months later, identify the baseline predictors of change in QOL.	Hospital database	Prospective observational	-	Italy	High income	151
Harmsen et al. (2020)	Evaluate the anticipation and the fulfilment of sexual activity after TKA in men and women and identify prognostic factors.	Hospital databases	Prospective cohort	KOOS; SF-12; EQ-5D; EQ_VAS	The Netherlands	High income	866
Hafkamp et al. (2020b)	Examine the relationship between physicians’ expectations patients’ expectations and primary outcome measures.	Hospital database	Prospective cohort	HSS-KRES; KOOS	The Netherlands	High income	190
Hafkamp et al. (2020a)	Identify and characterise different subgroups of osteoarthritis patients with respect to the amount and level of expectations.	Hospital database	Prospective cohort	HSS-KRES; KOOS	The Netherlands	High income	156
Tolk et al. (2020)	Analyse the relationship between preoperative factors and preoperative outcome expectations in TKA patients.	Hospital database	Cross-sectional	KOOS-PS; OKS; HSS-KRES; EQ-5D; EQ-VAS	The Netherlands	High income	204
Hafkamp et al. (2021)	Examine different trajectories of physical symptoms in patients with preoperative anxiety and depressive symptoms.	Hospital database	Prospective cohort	WOMAC; KOOS	The Netherlands	High income	186
Van Egmond et al. (2021)	Distinguish specific recovery patterns using the Oxford knee score (OKS) to explore predictors of less favourable recovery patterns.	National registry	Descriptive retrospective	OKS; EQ-5D	The Netherlands	High income	809
Leichtenberg et al. (2021)	Investigate if OA-associated pain, functional limitations and QoL are associated with objectively measured physical activity in patients with end-stage hip/knee OA.	Hospital database	Cross-sectional cohort	SF12; KOOS	The Netherlands	High income	48
Hoelen et al. (2024)	Determine the association between socioeconomic status (SES) and patient-reported outcome measures.	National registry	Retrospective review	KOOS-PS; EQ-5D-3L; EQ-VAS	The Netherlands	High income	78 811
Lindberg et al. (2021b)	Identify subgroups of patients with distinct chronic pain profiles following TKA and identified preoperative characteristics associated with these profiles.	Hospital database	Longitudinal cohort	-	Norway	High income	202
Getachew et al. (2021)	Test the hypothesis that the co-occurrence of high symptom levels prior to surgery is a risk factor for pain 12 months after TKA.	Hospital database	Longitudinal cohort	-	Norway	High income	202
Lindberg et al. (2021a)	Identify subgroups of patients with distinct pain profiles for 12 months following TKA.	Hospital database	Longitudinal cohort	-	Norway	High income	245
Kocic et al. (2020)	Evaluate patients’ perception of function and physical and mental dimensions of health-related quality of life (HRQoL).	Hospital database	Cross-sectional	OKS; SF-36v2	Serbia	Upper middle income	100
Jiménez Ortiz et al. (2020)	Analyse the influence of preoperative anxiety and depression on TKA outcomes.	Hospital database	Longitudinal observational prospective	WOMAC; KSS	Spain	High income	260
Dursteler et al. (2021)	Investigate whether preoperative CPM can predict persistent pain after KR surgery	Hospital database	Retrospective cohort	WOMAC; SF-36	Spain	High income	146
Sebastia- Forcada et al. (2024)	Assess clinically important differences in functional outcome over 10 years after primary total knee arthroplasty.	Hospital database	Prospective observational cohort	WOMAC; KSS; (SF)-12	Spain	High income	309
Berg et al. (2020)	Study the influence of fast-track programmes on patient-reported outcomes (PROs) 1 year after surgery.	National registry	Retrospective cohort	EQ-5D; EQ VAS; KOOS	Sweden	High income	8393
Mahdi, Halleberg-Nyman and Wretenberg (2020)	Investigate the prevalence of symptom improvement among patients with preoperative anxiety and/or depression.	National registry	Prospective cohort	KOOS; EQ-5D-3L	Sweden	High income	458
Mahdi, Halleberg-Nyman and Wretenberg (2021)	Investigate changes in the prevalence of anxiety and depression 1 year after primary TKA.	Hospital database	Prospective cohort	KOOS; EQ-5D-3L	Sweden	High income	403
Giesinger et al. (2020)	Comparison of pre-surgery patient characteristics (sex, age, BMI, comorbidity), health status and joint awareness.	National registry	Retrospective analysis	FJS-12; EQ-5D-3L	Switzerland	High income	2075
Huber et al. (2021)	Differences in the suitability for KA between different knee morphotypes and the differences between a true KA and an rKA in terms of alignment.	Hospital database	Retrospective review	KOOS; KSS; OKS, EQ-5D	Switzerland	High income	111
Giesinger et al. (2021)	Investigate the impact of body mass index (BMI) on improvement in pain, function and general health status following total knee arthroplasty.	Hospital database	Retrospective analysis	WOMAC; EQ-5D-3L	Switzerland	High income	1565
Vogel et al. (2024a)	Evaluate the responsiveness of different patient-reported outcome measures in patients with primary total knee arthroplasty.	Hospital database	Prospective observational	KSS; KOOS; FJS-12; EQ-5D-3L	Switzerland	High income	309
Vogel et al. (2024b)	Correlation of preoperative patient expectations with postoperative satisfaction, patient characteristics or patient-reported outcome measures.	Hospital database	Retrospective observational	HSSK-RES; EQ-5D-3L; EQ-VAS	Switzerland	High income	193
Kaya et al. (2024)	Determine the relationship between preoperative knee joint function and postoperative QoL in patients undergoing primary TKA.	Hospital database	Cross-sectional descriptive	OKS; EQ-5D-5L; EQ-VAS	Turkey	Upper middle income	208
Clement et al. (2020)	Assess a clinically important change in the Oxford knee score (OKS) between one and 2 years postarthroplasty, and identify predictors of change.	Hospital database	Retrospective cohort	OKS; EQ-5D	United Kingdom	High income	5857
Giesinger et al. (2020)	Comparison of pre-surgery patient characteristics (sex, age, BMI, comorbidity), health status and joint awareness.	National registry	Retrospective analysis	FJS-12; EQ-5D-3L	United Kingdom	High income	994
Ramaskandhan et al. (2020)	Study the 1-, 3-\ and 5-year outcomes of TAR in comparison with the THR and TKR outcomes.	Hospital database	Prospective cohort	WOMAC; SF-36	United Kingdom	High income	3520
Evans et al. (2021)	Describe the association of BMI at the time of surgery with revision after 10 years, 90-day mortality and patient-reported outcomes 6 months following primary.	National registry	Observational retrospective cohort	OKS	United Kingdom	High income	490 351
Mohammad, Judge and Murray (2021)	Compared the functional outcomes and quality of life of matched TKRs and UKRs, both overall and in different age groups, using data from 3 national datasets.	National registries	Retrospective observational	OKS; EQ-5D	United Kingdom	High income	254 355
Ramaskandhan et al. (2021)	Evaluate PROMs only and does not include clinical, radiographic or adverse event outcomes from surgery.	Hospital database	Prospective	SF-36; WOMAC	United Kingdom	High income	2475
Scott et al. (2021)	Determine association of radiographic severity, extent or pattern of knee OA with pain and function before total knee arthroplasty (TKA) or improvement therein 1 year after TKA.	Hospital database	Cross-sectional	OKS; EQ-5D-3L	United Kingdom	High income	259
Fabiano et al. (2024)	Exploratory analysis to examine how physical activity and HRQoL evolve in a defined cohort of patients with post-operative chronic pain over the initial 12 months post-TKR period.	Hospital database	Secondary to a randomised controlled trial	OKS-PS; EQ-5D-5L	United Kingdom	High income	83
**Articles from the Middle East region (*n* = 3)**							
Moghtadaei et al. (2020)	Investigate the influence of psychological status and physical and mental health on the outcome of patients undergoing TKA.	Hospital database	Prospective cross-sectional	SF-12; KOOS	Iran, Islamic Rep.	Upper middle income	52
Al-Otaibi (2021)	Analyse and discuss patient characteristics and their outcomes in the Abha region of Southwestern Saudi Arabia.	Hospital database	Cross-sectional	KSS	Saudi Arabia	High income	420
Alomran (2022)	To investigate the QOL and overall satisfaction post TKA.	Hospital database	Retrospective review	WOMAC; SF-36	Saudi Arabia	High income	200
**Articles from the North America region (*n* = 33)**							
King et al. (2020a)	Assess prior use of core recommended non-surgical treatment among patients with knee osteoarthritis (OA) scheduled for total knee arthroplasty (TKA).	Regional registry	Cross-sectional prospective cohort	WOMAC; KOOS-PS	Canada	High income	2277
King et al. (2020b)	Assessed the association between comorbidity and use of recommended OA therapies with current opioid use.	Regional registry	Cross-sectional prospective cohort	WOMAC; KOOS-PS	Canada	High income	2769
Lebedeva et al. (2021)	Evaluate resource use, costs and health-related quality of life (HRQoL) across the continuum of care for patients with knee OA.	Hospital database	Prospective cohort	WOMAC; SF-12; EQ-5D-5L	Canada	High income	119
Perruccio et al. (2020)	Investigate whether sex modified the influence of presurgery characteristics on post-TKA knee pain.	Hospital database	Prospective	KOOS-PS	Canada	High income	477
Costello et al. (2021)	Discover associations between a number of demographic, anthropological, epidemiological and medical factors and non-responders to TJR.	Regional registry	Prospective cohort	WOMAC	Canada	High income	416
Baghbani- Naghadehi et al. (2022)	Evaluate the association between BMI, patient-reported outcome measures (PROMs) preoperatively, and 3 and 12 months postoperatively.	Regional registry	Retrospective secondary analysis	WOMAC; EQ-5D	Canada	High income	7714
Christensen et al. (2021)	Identify which patient characteristics are related to gait mechanics in the surgical limb during walking post-TKA.	Hospital database	Cross-sectional	KOS-ADL	United States	High income	191
Anastasio et al. (2020)	Investigate potentially modifiable risk factors affecting LOS, with a focus on opioid use.	Hospital database	Cross-sectional	-	United States	High income	1033
Kunze et al. (2020)	Develop machine learning algorithms to predict dissatisfaction after TKA.	Hospital database	Retrospective review	PRHS; KSS	United States	High income	430
Lange et al. (2020)	Identify specific and disparate trajectories of PROs in the first year following TKA and correlate with patient characteristics.	Hospital database	Prospectivecohort	SF-36; KOOS	United States	High income	656
Lopez-Olivo et al. (2020)	Evaluate the association of preoperative psychosocial and demographic factors with total knee arthroplasty (TKA) outcomes.	Hospital database	Prospectivecohort	WOMAC; SF-36	United States	High income	178
Riddle (2020)	Examine osteoarthritis and symptom severity profiles of index versus contralateral knees of persons preparing for KA.	Hospital database	Cross-sectional	WOMAC	United States	High income	362
Sauder et al. (2020)	Investigate differences in patient demographics between centres in the United States, Scandinavia and South Korea for patients undergoing primary TKA.	Hospital databases	Cross-sectional	KOOS	United States	High income	129
Haffar et al. (2021)	Investigate the relationship between patient resilience, mental health and functional outcomes, and satisfaction up to 2 years after primary unilateral TKA.	Hospital database	Prospectivecohort	KOOS, Jr.; KSS; VR-12	United States	High income	86
Johnson et al. (2021)	Document the nationwide trends in age and obesity in primary THA and TKA throughout the obesity epidemic.	Hospital database	Retrospective analysis	-	United States	High income	1 556 651
Melnic et al. (2021)	Investigate the relationship between patient-reported mental health and postoperative physical function following TKA.	Hospital databases	Retrospectivereview	KOOS-PS	United States	High income	1392
Sideris et al. (2021)	Analyse the relationship between persistent postoperative pain scores and perioperative cytokine levels in patients undergoing unilateral TKA.	Hospital database	Prospective cohort	-	United States	High income	162
Emara et al. (2021)	Test the association of preoperative overdose risk score with postoperative health care use.	Hospital database	Prospective cohort	-	United States	High income	4326
Dugdale et al. (2021)	Learn more about real-world patient experiences in returning to driving after total knee arthroplasty.	Regional registries	Retrospectivereview	-	United States	High income	541
Darrith et al. (2021)	Determine which patient demographic factors influence the postoperative Patient-Reported Outcomes Measurement Information System (PROMIS) Global Health (GH) scores.	Hospital database	Retrospectivecohort	KOOS-JR	United States	High income	872
Katakam et al. (2021)	Assess the association between body mass index (BMI) and failure to achieve the 1-year Knee Disability and Osteoarthritis Outcome Score-Physical Function Short Form (KOOS-PS).	Regional registries	Retrospectivereview	KOOS-PS	United States	High income	1059
Held et al. (2022)	Investigate perioperative outcomes, complications and early patient-reported outcome measures (PROMs) of one imageless RA-TKA system compared to conventional.	Hospital database	Retrospectivecohort	KSS-FS; WOMAC; SF-12	United States	High income	221
Kugelman et al. (2022)	Understand the use of opioids during the acute post-surgical episode following TJA.	Hospital database	Retrospectivereview	-	United States	High income	5784
Zhai et al. (2023)	Determine overall 30-day mortality rate for unilateral primary elective TKA patients, stratified by age, comorbidities and preoperative diagnosis.	Hospital database	Retrospective analysis	-	United States	High income	325 837
Spiering et al. (2024)	Assess the responsiveness and determine the minimally important difference of 2 patient-reported outcome measures (PROMs) in patients after TKA.	Hospital database	Retrospectivecohort	KOOS-JR; PROMIS	United States	High income	1315
Gebauer et al. (2024)	Is baseline depression diagnosis associated with the likelihood of and time to TKA.	Regional registries	Retrospectivecohort	-	United States	High income	9466
Rechenmacher et al. (2024)	Examine the impacts of preoperative weight loss on patient-reported and adverse outcomes among TKA patients.	Hospital database	Retrospective analysis	PROMIS	United States	High income	90
Blackburn et al. (2024)	Determine the 1-year postoperative factors, specifically patient-reported outcome measures (PROMs) that were associated with 3-year and 5-year postoperative satisfaction.	Hospital database	Retrospectivereview	-	United States	High income	404
Benes et al. (2024)	Determine 1-year postoperative factors associated with 3-year and 5-year postoperative satisfaction.	National registries	Retrospectivereview	KOOS; EQ-5D-3L; EQ-5D-VAS	United States	High income	270
Albright et al. (2024)	Determine if Knee Injury and Osteoarthritis Outcome Score Joint Replacement (KOOS-JR) MCID values varied among patients undergoing TKA based on patient-specific factors.	Hospital databases	Retrospective review	KOOS-JR; PROMIS	United States	High income	976
Rahman et al. (2024)	Investigate the relationship between outcomes following total knee arthroplasty (TKA) and both the Social Vulnerability Index (SVI) and the Area Deprivation Index (ADI).	Regional registry	Retrospective review	KOOS, JR	United States	High income	19 321
Zheng et al. (2024)	Explore adding a single variable, the preoperative PROM score, to the CMS-Yale model would improve its ability to predict TKA success.	Hospital database	Prospective cohort	KOOS, JR; SF-36MCS	United States	High income	5958
Kop et al. (2024)	Determine the prevalence of contralateral joint OA for patients presenting for unilateral total knee (TKA).	Hospital database	Retrospective review	-	United States	High income	933
Jabbouri et al. (2024)	This study evaluates trends of cemented versus press-fit total knee arthroplasty (TKA).	National registry	Retrospective review	-	United States	High income	348 282
**Articles from the South Asia region (*n* = 4)**							
Devasenapathy et al. (2020)	Compare impairments, activity limitation and participation restriction between men and women scheduled for TKA.	Hospital database	Cross-sectional analysis	KOOS; LEAS; LLDI	India	Lower middle income	240
Chhaya et al. (2020)	Evaluate the quality-of-life following physiotherapy management in patients with total knee replacement.	Hospital database	Cross-sectional	EQ-5D; KOOS	India	Lower middle income	11
Bakshi et al. (2021)	Assess the HRQOL outcome after TKR in patients with knee osteoarthritis (OA).	Hospital database	Longitudinal cohort	KOOS	India	Lower middle income	56
Chaudhary et al. (2024)	Assess early functional and clinical outcomes of TKA by analysing post-operative mechanical axis and Knee Society Score (KSS) data.	Hospital database	Prospective observational	KSS	India	Lower middle income	40

Note: Please see the full reference list of the article, Coetzee, M., Clifford, A., Leibbrandt, D.C., Jordaan, J. & Louw, Q., 2026, ‘The global profile of individuals undergoing total knee replacement surgery through a PROGRESS-PLUS equity lens: A systematic review’, *South African Journal of Physiotherapy* 82(1), a2303. https://doi.org/10.4102/sajp.v82i1.2303, for more information.

WOMAC, Western Ontario and McMaster Universities arthritis index; SF 36, 36-item short form health survey; SF 12, 12-item short form health survey; OKS, Oxford knee score; KSS, knee society score; EQ-5D-5L, European quality of life 5 dimensions 5 level; KOOS, knee injury and osteoarthritis outcome score; KOOS JR, knee injury and osteoarthritis outcome score; joint replacement; KOOS PS, knee injury and osteoarthritis outcome score; short form; HSS, hospital for special surgery knee rating scale; VR 12, The veterans rand 12 item health survey; PROMIS, patient reported outcomes measurement information system; FJS, forgotten joint score; BMI, body mass index; PRO, patient reported outcome measures; LOS, length of stay; HRQoL, health-related quality of life; LLDI, late-life disability instrument; LEAS, levels of emotional awareness scale.

Table 4 summarises the PROGRESS-Plus data extracted from the studies, and Table 5 summarises the PLUS factors such as smoking, BMI and severity. When combining the results of different studies for one country, the range of the results is reported. Sample sizes varied widely, ranging from 11 to over 490 000 participants, and the final sample of individuals included in our review is 3 203 292. Australia, Denmark, the United Kingdom (UK) and the United States (US) used large knee replacement databases for their studies and were able to provide retrospective data on basic demographic profiles of their OA population. Most studies employed retrospective or prospective hospital databases and national or regional registries, with a smaller number using cross-sectional, descriptive or longitudinal designs. Common data collection instruments included validated patient-reported outcome measures (PROMs), reflecting a focus on functional recovery, patient satisfaction, mental health, pain and quality of life post-TKR.

**TABLE 4:** Summary of the PROGRESS-Plus factors from all the countries included (2020–2024) according to the income level of the country.

Country	Number of studies	Sample total (*n*)	Age (years) (Mean range)	Gender Female		Race† White		Occupation† employed		Occupation† pension		Education† Secondary		SES† Low		Social capital† partner	
				*n*	%	*n*	%	*n*	%	*n*	%	*n*	%	*n*	%	*n*	%
**High income**																	
Australia	3	10 204	67–68.5	5853	57.4	-	-	-	-	-	-	-	-	-	-	-	-
Belgium	1	280	66.7	152	54.0	-	-	-	-	-	-	165	-	-	-	-	-
Canada	6	13 772	65.4–66.9	7104	51.6	-	-	33	27.7	75	63.0	555	24.0	643	26.8	95	80.0
Denmark	4	26 069	64–68	15 578	59.76	-	-	-	-	-	-	-	-	-	-	-	-
Germany	1	63	72.1	41	65.0	-	-	-	-	-	-	-	-	-	-	-	-
Hong Kong	1	1566	68–69	1170	75.0	-	-	-	-	-	-	-	-	-	-	-	-
Italy	1	151	67.9	97	64.0	-	-	-	-	-	-	46	30.0	-	-	-	-
Japan	4	1338	73.6–74.9	1112	83.2	-	-	-	-	-	-	-	-	-	-	-	-
Korea	6	1703	68–72.8	1357	79.7	-	-	-	-	-	-	-	-	-	-	-	-
The Netherlands	8	81 270	65–70	50 843	62.6	-	-	127	21.9	-	-	357	65.4	-	-	768	73.0
New Zealand	1	214	68.5	101	47.0	185	86.0	-		-	-	-	-	-	-	-	-
Norway	3	649	68–68.3	409	63.0	202	100.0	259	57.9	-	-	325	50.1	-	-	407	62.7
Saudi-Arabia	2	620	62.7–69.5	498	80.3	-	-	-		-	-	-	-	-	-	-	-
Singapore	2	3000	66.3–68	2115	70.5	-	-	-		-	-	-	-	1988	66.3	-	-
Spain	3	715	69.2–73.1	497	69.5	-	-	-		-	-	-	-	-	-	-	-
Sweden	3	9254	69–70	5250	56.7	-	-	-		-	-	-	-	-	-	-	-
Switzerland	5	4253	68–69.3	2723	63.9	1676	100.0	293	18.7	730	46.7	1537	98.0	-	-	-	-
Taiwan	1	357	71	282	79.0	-	-	-	-	-	-	-	-	-	-	-	-
UK	9	757 945	66.8–70.3	434 350	57.3	243 452	96.0	-	-	-		-	-	-	-	-	-
US	29	2 287 146	63–69.5	1 405 929	61.5	1 852 834	81.2	77	43.3	85	47.8	753	68.2	3801	17.7	1283	68.7
**Upper middle income**																
China	5	1699	65.3–75.5	1338	78.8	-	-	-	-	-	-	167	50.0	-	-	197	94.0
Georgia	1	197	70.2	-	-	-	-	-	-	-	-	-	-	-	-	-	-
Iran	1	52	67.2	37	73.0	-	-	-	-	-	-	-	-	-	-	-	-
Serbia	1	100	69.4	69	69.0	-	-	6	6.0	69	69.0	-	-	-	-	94	94.0
Turkey	1	208	65.7	179	86.0	-	-	8	4.0	-	-	22	10.6	-	-	162	78.0
**Lower middle income**																
India	4	347	58.9–65.6	236	68.0	-	-	47	20.0	-	-	105	43.8	-	-	101	42.1
Philippines	1	111	64.5	93	84.0	-	-	-	-	-	-	-	-	-	-	-	-

SES, Socioeconomic status.

† , Does not include the frequency from studies with no data on the variable.

**TABLE 5:** Summary of the Plus factors, including smoking, body mass index and osteoarthritis severity, from all the countries included (2020–2024) according to the income level of the country.

Country	Number of studies	Smoking Yes†		BMI(Mean range)†	OA severity KL IV†		ASA II†		ASA III†		Previous surgery†	
		*n*	%		*n*	%	*n*	%	*n*	%	*n*	%
**High income**												
Australia	3	-	-	32.5	-	-	4443	53.5	3379	40.7	-	-
Belgium	1	-	-	29.3	30	10.7	-	-	-	-	97	34.6
Canada	6	177	6.4	30.7–34.9	-	-	-	-	-	-	-	-
Denmark	4	-	-	28.9–29.5	42	24.9	-	-	-	-	5421	21.6
Germany	1	-	-	30.4	-	-	-	-	-	-	-	-
Hong Kong	1	-	-	27.1–28.4	-	-	-	-	-	-	-	-
Italy	1	17	11.3	28.7	-	-	-	-	-	-	-	-
Japan	4	-	-	25.6–27.2	575	62.2	-	-	-	-	-	-
Korea	6	76	8.5	25.6–27.1	1014	67.5	161	80.5	33	16.5	-	-
The Netherlands	8	6278	7.9	29–30.6	177	86.8	52 511	66.6	16 462	20.9	-	-
New Zealand	1	-	-	30.1	-	-	147	68.7	40	18.7	-	-
Norway	3	-	-	28–29.4	-	-	-	-	-	-	-	-
Saudi Arabia	2	-	-	28.4–29.4	-	-	-	-	-	-	-	-
Singapore	2	-	-	27.3–28	-	-	-	-	-	-	-	-
Spain	3	-	-	30.8–31.2	-	-	-	-	-	-	-	-
Sweden	3	-	-	28.8–29	-	-	565	65.6	72	8.3	-	-
Switzerland	5	269	17.2	29.6	394	78.5	378	75.3	92	10.3	61	55.0
Taiwan	1	20	5.6	28	152	42.6	-	-	-	-	-	-
UK	9	3	5	27.9–30.9	142	54.8	552 926	74.2	123 259	16.6	-	-
USA	29	2241	8.3	29.8–36.7	246	50.1	12 016	42.5	15 179	53.7	66	40.7
**Upper middle income**												
China	5	-	-	23.5–30	-	-	304	73.8	53	12.9	-	-
Georgia	1	-	-	30.2	-	-	61	-	39	19.8	-	-
Iran	1	-	-	-	-	-	-	-	-	-	-	-
Serbia	1	-	-	29	-	-	-	-	-	-	-	-
Turkey	1	3	1.4	32.1	-	-	-	-	-	-	-	-
**Lower middle income**												
India	4	-	-	28.7	-	-	-	-	-	-	-	-
Philippines	1	-	-	-	-	-	-	-	-	-	-	-

BMI, body mass index; ASA, American Society of Anaesthesiologists Classification system.

† , Does not include the frequency from studies with no data on the variable.

Across all income levels, the majority of study participants were female, typically ranging from 54% to 86%. The mean age of participants ranged between 65 years and 75 years. Some variations across countries, such as Georgia, Germany and Japan, which had an older average age than India, which had the youngest cohort. Information on race/ethnicity was limited, with only a few countries reporting data on the percentage of white participants. Data on occupation, education and SES were sparse, particularly for lower-income countries. In HICs, such as Canada, the Netherlands and the US, where data were available, a significant proportion of participants had secondary education or higher (ranging from 24% to 98%) and were employed or receiving pensions. Low SES was reported for 17.7% of participants in the US and 26.8% in Canada. Social capital, measured by the percentage of participants with a partner, was reported only for a few HICs, with the highest percentage (94%) observed in China.

Smoking prevalence was reported for a few HICs, ranging from 5% to 17.2% of participants. Mean BMI tended to be higher in HICs (ranging from 25.6 kg/m^2^ to 36.7 kg/m^2^) compared to upper-middle and lower-middle-income countries (23.5 kg/m to 32.1 kg/m). Osteoarthritis severity, assessed using the Kellgren–Lawrence (KL) grading system, was reported for some HICs. The percentage of participants with severe OA (KL grade IV) ranged from 10.7% in Belgium to 78.5% in Switzerland. American Society of Anaesthesiologists (ASA) physical status classification was reported for several HICs, with a substantial proportion of participants classified as ASA II (ranging from 42.5% in the US to 80.5% in Korea) or ASA III (ranging from 8.3% in Sweden to 53.7% in the US). Previous surgery was reported for a few HICs, with percentages ranging from 34.6% in Belgium to 55% in Switzerland.

## Discussion

The purpose of our review was to describe the demographic and health equity profiles of adults undergoing TKR for primary OA in lower, middle- and high-income countries. The review included 101 studies encompassing over 3.2 million individuals and represents the first comprehensive examination of the global demographic and health equity profile of adults undergoing TKR for primary OA through the PROGRESS-Plus equity lens.

Although a substantial volume of published research demonstrates ongoing clinical and academic interest in TKR, the findings show critical geographic biases and equity reporting deficiencies that fundamentally limit the clinical utility of the current evidence base in different contexts.

The concentration of TKR research in high-income and upper-middle-income countries underscores a significant gap in understanding the diverse populations affected by OA. This lack of representation from low-income countries is a significant limitation, as this may affect the generalisability of findings and limit the understanding of how socioeconomic factors influence outcomes in TKR across diverse populations. For example, no African studies met the criteria for inclusion, and this is problematic as the health system-related factors may significantly impact access to and quality of OA care in African contexts. Africa contributes significantly to the increasing global prevalence (estimated more than 37%), unmet needs and treatment burden for people with OA (Steinmetz et al. 2023). Despite the availability of evidence-based practice recommendations for OA management, evidence suggests that the implementation of these guidelines among African health professionals remains poor (Keller & Sankah 2025). Individuals often report a lack of support from clinics, with treatments predominantly focusing on pharmacological management (painkillers) and minimal mention of rehabilitation or pain management (Keller & Sankah 2025). There is an urgent need for a system-wide initiative that prioritises comprehensive, affordable and evidence-based OA care in low-resource settings, including improved education, consistent care pathways and better access to a range of appropriate health professionals and facilities (Owoyemi et al. 2025).

The application of the PROGRESS-Plus framework showed systematic underreporting of social determinants of health across all income levels, although particularly pronounced in lower-income countries. Equity indicators were inconsistently documented, limiting understanding of how these factors shape TKR access, outcomes and recovery trajectories.

This reporting gap is not merely a methodological oversight but represents a fundamental barrier to addressing documented health inequities. Without comprehensive equity data, service planning cannot be appropriately tailored, interventions risk perpetuating existing disparities, and the mechanisms through which social determinants influence clinical outcomes remain unsolved (Rizvi et al. 2022). Data pertaining to occupation, education and SES were particularly scarce, especially in the studies originating from lower-income countries. This is in contrast with the HICs such as Canada, the Netherlands and the US, which reported more comprehensive data on socioeconomic factors such as education and employment status. In these regions, 24% to 98% of participants undergoing TKR had attained secondary education or higher, and the majority of participants were either reemployed or receiving a pension (Ashkenazi et al. 2024). However, low SES was still a concern among this population, with figures indicating that approximately 17.7% of participants in the US and 26.8% in Canada were classified as having low SES. Social capital, which was most commonly defined as the percentage of participants with a partner, was reported in only a few HICs. In the included studies, China exhibited the highest percentage at 94%. This suggests a potential correlation between social support and health outcomes in OA populations (Wylde et al. 2019).

Despite geographic and methodological variation, the demographic profile demonstrated remarkable consistency, showing that individuals awaiting and undergoing TKR comprise 54% – 86% females, with a mean age consistently between 65 years and 75 years. This female predominance aligns with established literature attributing elevated OA burden in postmenopausal women to declining oestrogen levels, age-related degenerative changes and heightened pain sensitivity (Xu et al. 2025). However, this consistency contrasts with the clinical profile heterogeneity, particularly regarding BMI. The tendency towards higher mean BMI in HICs (25.6 kg/m^2^ – 36.7 kg/m^2^) compared with upper-middle and lower-middle-income countries (23.5 kg/m^2^ – 32.1 kg/m^2^) likely reflects differing lifestyle factors, dietary patterns and obesity prevalence (World Health Organization 2023). This variation underscores the necessity for context-specific pre-operative interventions, particularly weight optimisation programmes delivered by physiotherapists. The wide variation in reported severe OA (KL grade IV: 10.7% in Belgium to 78.5% in Switzerland) further suggests differing thresholds for surgical intervention, access barriers or disease progression patterns across settings.

### Strengths and limitations

This systematic review is the first review to look at a global profile of individuals undergoing TKR surgery. Furthermore, the use of the PROGRESS-PLUS framework for data extraction in our study underscores an equity lens that may not be consistently applied in other research.

This method of portraying a wider range of the social determinants of health is crucial for understanding health outcomes. It also highlights critical gaps in representation and the need for improved reporting practices in the field. Our study adhered to reporting guidelines, such as STROBE and PRISMA, to ensure clarity and consistency in published studies.

The reporting quality of the final list of included studies was assessed using the appropriate study design appraisal tool from the STROBE, and the PRISMA checklist for reporting systematic reviews was completed and is presented in Online Appendix 1 – Table 4-A1.

Despite the overall large sample size, the limited representation from low-income countries is a significant limitation. The absence of data from these regions restricts our understanding of how socioeconomic factors influence TKR outcomes. Because of the search being limited to studies published after 2020, studies published in Africa may have been missed. In addition, a meta-analysis of the data was not possible because of the heterogeneity in outcome measures used and the reporting methods; therefore, our study could only report on the similarities and differences between countries and reference the range of reported data.

### Implications for rehabilitation and physiotherapy

The implications of our review for rehabilitation are significant, particularly with regard to enhancing individualised management and addressing health equity in individuals undergoing TKR. Our study found significant gaps in the reporting and consideration of socioeconomic and equity-related information, especially in LMIC. This highlights the need for rehabilitation professionals involved in the management of individuals undergoing TKR, such as physiotherapists, to include socioeconomic indicators in pre-operative assessment, as this will assist with the tailoring of post-operative support and enhancing in postoperative recovery (Clynes et al. 2019). These indicators could include factors such as income, education and employment status. The reporting of demographic variables is essential to illustrate how social determinants of health influence pain, function and quality-of-life outcomes after TKR and is important to inform equitable service planning in the future management of these individuals (Castro et al. 2021; O’Neill et al. 2014).

In addition, physiotherapists are well positioned to implement recommended pre-operative lifestyle interventions such as education on exercise, weight optimisation and other self-management strategies (Rice et al. 2019). Incorporating interventions into the care pathway could improve postoperative outcomes by addressing modifiable risk factors and preparing individuals for surgery and recovery within their social context. More research within the field of physiotherapy should investigate the efficacy of these pre-operative lifestyle interventions on postoperative outcomes. This could provide insights into how pre-operative health behaviours influence recovery and overall satisfaction with surgical outcomes.

### Implications for physiotherapy practice, policy and research

Our review identifies critical evidence gaps with direct implications for physiotherapy practice, health equity, and service delivery for individuals undergoing TKR.

#### Physiotherapy practice: Equity-informed, biopsychosocial rehabilitation

The substantial underreporting of SES and equity indicators, particularly in LMICs, could potentially impact evidence-informed rehabilitation and patient outcomes for individuals awaiting or undergoing TKR.

Physiotherapists should systematically integrate socioeconomic indicators (income, education, employment status, housing stability) into pre-operative assessments to enable individualised care planning (Clynes et al. 2019). These data constitute essential clinical information, as social determinants fundamentally shape pain experiences, functional recovery and quality-of-life outcomes following TKR (Castro et al. 2021; O’Neill et al. 2014).

The findings highlight the importance of a biopsychosocial, equity-focused rehabilitation approach that explicitly accounts for socioeconomic and cultural contexts, especially in underrepresented LMIC populations, where comorbidity burdens, healthcare access barriers and socioeconomic disadvantage affect post-surgical recovery (Alvarez 2022). The observed higher baseline BMI in HICs reinforces the clinical relevance of weight optimisation, although the absence of African and broader LMIC data prevents assumptions about optimal strategies across diverse contexts.

In addition, the effectiveness of pre-operative physiotherapy interventions on post-TKR outcomes remains inadequately evaluated. Research must rigorously examine how pre-operative health behaviours, delivered within culturally appropriate frameworks, influence recovery, functional outcomes and patient satisfaction across diverse socioeconomic and geographic populations.

#### Policy implications: Addressing structural inequities

The geographic concentration of evidence reflects systemic research inequities requiring policy intervention. Research funding bodies must prioritise studies in LMICs and underrepresented settings to comprehensively understand TKR outcomes and develop contextually appropriate care pathways. Current evidence cannot support confident translation to populations facing substantially different socioeconomic circumstances and healthcare systems. Standardised reporting of health equity metrics should become mandatory in orthopaedic and rehabilitation research. The PROGRESS-Plus framework (place of residence, race or ethnicity, occupation, gender, religion, education, SES, social capital) provides validated structure for equity-focused data collection (O’Neill et al. 2014). Without such data, equitable service planning remains unattainable. Low-resource settings require system-wide reform where OA care remains fragmented and pharmacologically focused. Policy must support comprehensive, evidence-based care models ensuring equitable access to appropriately trained health professionals, including physiotherapists, and necessary facilities. The disproportionate disability burden in LMIC populations requires coordinated action.

#### Research priorities

Future studies should prospectively collect comprehensive equity indicators, employ standardised outcome measures and intentionally recruit diverse populations. Only through addressing these methodological imperatives can the evidence base support equitable, effective TKR care globally.

## Conclusion

This systematic review of 101 studies (including over 3.2 million individuals) highlights that while abundant research exists on the population of patients awaiting or undergoing TKR, the evidence base remains geographically concentrated in high-income and upper-middle-income countries, with a complete absence of African representation and profound underreporting of health equity indicators across all settings. This limits the generalisability of clinical guidelines to populations with the greatest disability burden from knee OA. Addressing this disparity requires coordinated action from researchers, who should systematically collect and report equity metrics, and policymakers, who should prioritise research funding for lower-income countries and mandate standardised equity reporting. Physiotherapists should adopt biopsychosocial, equity-focused rehabilitation approaches that integrate socioeconomic indicators into clinical assessment and deliver contextually appropriate pre-operative interventions.
